# Genome Sequence of *Saccharomyces cerevisiae* Strain Kagoshima No. 2, Used for Brewing the Japanese Distilled Spirit Shōchū

**DOI:** 10.1128/genomeA.01126-17

**Published:** 2017-10-12

**Authors:** Kazuki Mori, Chihiro Kadooka, Chika Masuda, Ai Muto, Kayu Okutsu, Yumiko Yoshizaki, Kazunori Takamine, Taiki Futagami, Hisanori Tamaki

**Affiliations:** a Computational Bio-Big Data Open Innovation Laboratory, National Institute of Advanced Industrial Science and Technology, Tokyo, Japan; b Education and Research Centre for Fermentation Studies, Faculty of Agriculture, Kagoshima University, Kagoshima, Japan

## Abstract

Here, we report a draft genome sequence of *Saccharomyces cerevisiae* strain Kagoshima no. 2, which is used for brewing shōchū, a traditional distilled spirit in Japan. The genome data will facilitate an understanding of the evolutional traits and genetic background related to the characteristic features of strain Kagoshima no. 2.

## GENOME ANNOUNCEMENT

*Saccharomyces cerevisiae* Kagoshima no. 2 is a diploid strain used for making shōchū, which is a traditional Japanese distilled spirit (1). Previously, *S. cerevisiae* strain Koshi no. 5 was isolated from awamori mash by Katsuta et al. in 1952 (2). Awamori is also a Japanese distilled spirit that uses rice as an ingredient. Later, strain Kagoshima no. 2 was isolated from Koshi no. 5 by Onoue in the late 1960s (2). Currently, more than 80% of shōchū distilleries in Kagoshima Prefecture, Japan, use strain Kagoshima no. 2 in shōchū production.

For the shōchū yeast strains, a high ethanol fermentation ability under relatively warm and low pH conditions is required because shōchū is produced mainly in a warm area in Japan; furthermore, shōchū mash contains a high concentration of citric acid produced by *Aspergillus luchuensis* or *A. luchuensis* mut. *kawachii* (3, 4). The citric acid reduces the pH to between 3 and 3.5, which prevents the growth of unexpected microbial contaminants during the fermentation process.

To better understand shōchū yeast, we sequenced the genome of strain Kagoshima no. 2, which was obtained from the stock culture collection at the Kagoshima Prefectural Institute of Industrial Technology. The genome of strain Kagoshima no. 2 was sequenced by a three-cell run on the PacBio RS II platform (Pacific Biosciences) and one paired-end run on the Genome Analyzer II (GAII) platform (Illumina) at Genaris Omics, Inc., and Hokkaido System Science Co., Ltd., respectively. The genomic DNA of strain Kagoshima no. 2 was sequenced to 324-fold and 73-fold coverages by PacBio RS II and GAII, respectively. The *de novo* assembly of the reads obtained by the PacBio RS II sequencer was performed using Canu version 1.3, and the reads obtained by GAII were used for error correction to generate 33 contigs, including an entire mitochondrial DNA contig. The draft genome of strain Kagoshima no. 2 comprises 12,317,859 bp with 5,637 predicted coding sequences and a GC content of 38.3%. The genome annotation of the obtained contigs was performed based on analysis with AUGUSTUS version 3.2.3 (5) and on BLAST searches against a nonredundant protein sequence database and the Saccharomyces Genome Database (http://www.yeastgenome.org).

Phylogenetic analyses using a multigene approach have shown that strain Kagoshima no. 2 is phylogenetically close to the *S. cerevisiae* sake yeast strains (6). Sake is a traditional Japanese alcoholic beverage made from yeast strains that also show a high alcohol fermentation ability. During a study of sake yeast, it was revealed that the mutation in the *RIM15* gene is involved in the high alcohol production ability of modern sake yeast strains (7, 8). In the genome of Kagoshima no. 2, a nonsense mutation was found in the *RIM15* gene, which may also be involved in the high alcohol production ability of shōchū yeast. Thus, the genome data of strain Kagoshima no. 2 will enhance our understanding of shōchū yeast.

### Accession number(s).

The nucleotide sequences of the chromosome of *S. cerevisiae* Kagoshima no. 2 have been deposited in GenBank/DDBJ under accession numbers BEGW01000001 to BEGW01000033.
